# SRAM-Based PUF Readouts

**DOI:** 10.1038/s41597-023-02225-9

**Published:** 2023-05-27

**Authors:** Sergio Vinagrero, Honorio Martin, Alice de Bignicourt, Elena-Ioana Vatajelu, Giorgio Di Natale

**Affiliations:** 1 Univ. Grenoble Alpes, CNRS, Grenoble INP, TIMA, 38000 Grenoble, France; 2 Electronic Technology Department, University Carlos III of Madrid, Madrid, Spain

**Keywords:** Characterization and analytical techniques, Software, Scientific data

## Abstract

Large-scale parameter characterization of Physical Unclonable Functions (PUFs) is of paramount importance in order to assess the quality and thus the suitability of such PUFs which would then be developed as an industrial-grade solution for hardware root of trust. Carrying out a proper characterization requires a large number of devices that need to be repeatedly sampled at various conditions. These prerequisites make PUF characterization process a very time-consuming and expensive task. Our work presents a dataset for the study of SRAM-based PUFs on microcontrollers; it includes full SRAM readouts along with internal voltage and temperature sensors of 84 microcontrollers of STM32 type. Data has been gathered with a custom-made and open platform designed for the automatic acquisition of SRAM readouts of such devices. This platform also provides possibilities of experimenting aging and reliability properties.

## Background & Summary

Physical Unclonable Functions (PUFs) have emerged in the last decade among the most cost-effective security primitives to be used as hardware trust anchor of many systems. Silicon PUFs exploit inherent manufacturing variations to generate sequences of bits that are not stored but produced upon request; thanks to the random and uncontrollable nature of such variations, the given sequences are uniquely generated for each device and therefore can be used as the circuit signature. These signatures are randomly distributed (among various devices and within the same device) and are stable within the same device at each request. They can be used in diverse secure applications, including the generation of secure keys, hardware identification, and challenge-response based authentication protocols. Among the manifold silicon PUFs proposed in the literature, the SRAM-PUF^1^ is one of the most popular because of the availability of its memory in every electronic device: due to transistors manufacturing variations, the symmetry of a SRAM cell can be broken, each cell having a preferred power-up state. Combining multiple cells leads to create start-up patterns suitable for signature.

Because of their paramount role in device security, PUFs must undergo exhaustive characterization of their properties in order to guarantee quality over lifetime and under various operating conditions. Such evaluation process is still subject to research, yet already typical widespread metrics are commonly employed in PUFs analysis^2^: *uniformity* is the distribution of Ones and Zeros in the start-up pattern; *reliability* is the variation of the start-up pattern on repeated power-on activation of the same device; *uniqueness* is the probability of having devices with different signatures; *bit-aliasing* is the probability of a specific bit position of the signature to be biased towards ‘0’ or ‘1’ over multiple devices.

The most challenging parameter to be assessed is *reliability*, since it requires many devices to be tested, during long periods of time, and under various environmental conditions. Traditionally, the influence of aging and operating conditions in PUF responses has been addressed by simulation^3^ or performing small datasets. Regarding SRAM-PUFs, special focus was given to SRAM embedded in microcontroller (*μ*C): in^1^, the authors collected 30 samples of 10 MSP430F1232 to study the PUF behaviour of that *μ*C; in^4^, they studied the main quality metrics of 26 STM32F303 and 31 STM32F407 collecting 37 samples for each *μ*C; in^5^, 200 samples of raw SRAM data from 144 Cortex-M4F *μ*Cs were published for further research on SRAM-PUFs. Most recently, the effects of aging has been thoroughly measured from 16 Arduino boards to get around 175 million measurements^6^.

The previous works have confirmed that SRAM-PUFs implemented in general purpose *μ*Cs could be used for most PUF applications. Nevertheless, small number of samples and devices (with the exception of^5^) limit further research touching spatial correlation, aging or suitable post-processing.

In this assignment we present a dataset for the study of SRAM-based PUFs on microcontrollers. It contains the Unique Identifier (UID) of each device stored in memory by the vendor at manufacturing process. This UID comprises the device batch number as well as information such as wafer number, wafer lot and position of the *μ*C. The details can be specifically useful when analyzing spatial correlation that could unveil unknown PUFs vulnerabilities^7^. Our custom-made platform gathers data and enables experiments on aging and reliability.

## Methods

All the collected data has been gathered thanks to this open platform designed from scratch at TIMA Laboratory. The requirements for its creation were the capacity to:

1. be able to gather data from a large number of devices in order to ensure statistically relevant results.
2. automatically power-cycle each device as characterizing SRAM-based PUFs involves such compulsory and time-consuming process.
3. guarantee data integrity, considering storage over time and transmission without corruption.
4. be scalable, to allow for future development on other microcontrollers of various vendors.

This platform has been designed to work with any microcontroller but the data has been assembled from 84 boards of STM32L152RE by ST Microelectronics. Moreover, it also provides easy access to a comprehensive database involving thousands of samples of multiple boards; it is of high interest since there are barely no dataset available to the public. Additionally, this platform offers advantages over ad-hoc solutions normally built for this purpose.

Our platform will save many resources to the users in terms of money (buying hundreds of devices) and time (collecting thousands of samples). The availability of raw-data will empower any user to carry out various experiments (e.g. designing new post-processing, searching for systematic variations, etc.) with a number of samples and devices big enough to consider the experiment statistically significant. Besides, the extra information provided (operating conditions, wafer position, etc.) will open a variety of options to detect vulnerabilities and develop new metrics. Additionally, this platform gives access to real-time core voltage and temperature of the *μ*C thanks to the measurements of sensors integrated on each *μ*C. Operating conditions have proven of paramount importance for the stability of PUF responses so the information provided by the on-chip sensors is necessary in any analysis.

As a totally new feature to the best of our knowledge, we offer the possibility to interact with the boards by controlling the On/Off switch time of microcontrollers (data remanence studies) and to write custom values in the SRAM (NBTI studies). One can write any value at any SRAM region facilitating NBTI effects. It is well-known that storing certain value in a SRAM cell (e.g.,0), reinforces the tendency of power-up to the opposite value (e.g.,1) due to aging mechanisms^8^. NBTI, standing for *Negative Bias Temperature Instability*, is a common phenomenon in PMOS transistors, increasing the threshold voltage and thus resulting in the decrease of drain current and transconductance. With time, inverters in the SRAM cells may not behave as initially set up. *Reliability* being the capacity of a PUF to produce the same response under various conditions and timeframes, in^9^ it is proposed to induce aging into the transistors in order to improve this reliability. For that purpose, the opposite value of the one originally obtained is written into the cells.

### Platform description

**Fig. 1:**
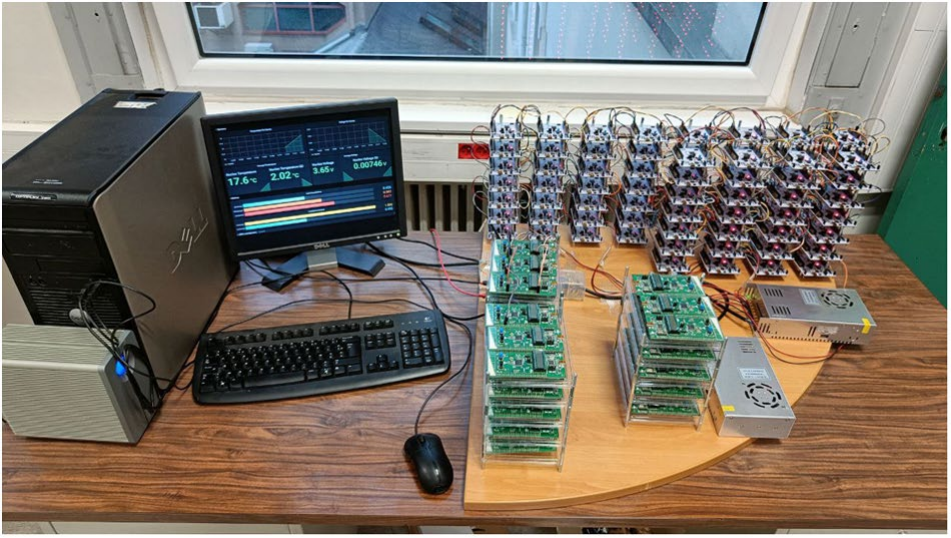
Picture of the deployed platform used to gather the data.

The platform comprises diverse components designed to work together in order to easily gather data from a vast amount of devices while ensuring its integrity. The code is built with focus on scalability to ensure the application of different devices without altering the main mechanics of the station. The platform receives instructions from a message broker, allowing for the necessary commands to be performed on the devices; data is stored in a SQL database; every process is monitored and logged in real time to flag unexpected behaviour or error. The station is depicted in Fig. 1.

The requests from the message broker are split into different atomic “commands”. They are queued so that multiple commands can be sent at the same time but be executed sequentially. A timeout for each command is also set to avoid hanging forever. Communication with the devices is performed through a defined interface that translates commands into necessary packets of custom-made communication protocol. This process makes the platform very scalable indeed, multiple devices can be connected at the same time as long as they all use the same communication interface.

#### Communication protocol

A packet-based communication protocol has been designed to transfer data between the PC and the devices. The packet contains bytes to transmit as well as metadata. Data is stored in the devices in a C struct, serialized when sending and deserialized when receiving. These packets are used to build *atomic* operations that will carry out the designed actions.

Table 1 presents the different atomic operations that the platform can perform. They are the basis of more complex commands Tables 1, 2.

**Table 1:** Available commands in the platform.

Command	Description
**ACK**	Acknowledgement of an operation. An ACK is sent by a device, for example after a WRITE operation to inform the PC that the command has successfully been executed.
**PING**	This command gives the PC the number of devices that are connected in a chain and their SRAM sizes.
**READ**	Read a region of information of a device. The Options field in the packet contains the offset to read from. The offset is the number of 512 bytes to skip. If the region cannot be read or the checksum does not match, an ERR is transmitted back to the station.
**WRITE**	Write a series of bytes in a memory region of a device. The body in the packet contains the bytes to write. An ERR is sent back to the station in case the checksum does not fit.
**INVERT**	This command employs the WRITE command to deliver the opposite values of the first sample.
**SENSORS**	Extract the sensors information from a device. Microcontrollers connected to the platform have temperature and core voltage sensors.
**LOAD**	Load source code to a device that would later be executed.
**EXEC**	Execute code loaded by the LOAD command and store the results into a circular buffer that can later be retrieved.
**RETR**	Retrieve results stored after the code has been executed.
**ERR**	Error during communication. It can be a wrong checksum or a problem during the parsing of a packet.

**Table 2:** Potential problems that may occur during a WRITE operation.

Log level	Information
**ERROR**	Serial port is off. Please turn on the serial port first
**ERROR**	No device managed
**ERROR**	Device {device} is not managed
**ERROR**	Offset {offset} for device {device} must be in range [0, {max_offset}]
**ERROR**	Writing problem in device {device} at offset {offset}
**ERROR**	Packet {packet} for device {device} is corrupted
**INFO**	Data written correctly

#### Custom code execution

As shown above, the platform provides commands for custom code exception. Code execution is performed thanks to zForth (https://github.com/zevv/zForth) being is a subset of Forth, designed to be highly portable. Each device comprises an instance of zForth interpreter that can read code from a buffer. As an example, the following code calculates the Fibonacci sequence from 1 to 1024.

: fib 1 1 begin.. dup rot rot + dup 1024 > until; fib

The same code can be executed multiple times without need of loading every instance. The results are automatically written in a circular buffer that can later be retrieved. Users will be able to exploit this functionality for their experiments: make a request that will be queued and once done, the data will be sent to them directly.

### Devices

In order to maximize the number of connections to the PC, devices are united in Daisy Chains and two UART ports are plugged to communicate with the rest of the devices in both directions. All the devices are programmed with the same source code, which makes the process of adding, removing and changing equipment fairly trivial. The physical position of each one is detected through a field in our communication protocol called PIC, standing for *Position In Chain*. The value starts at 0 at PC level and is incremented with 1 for every jump the packet performs downstream. This field along with the 96-bit identifier provided by the manufacturer stored in memory allows to fully know the position of a device in the chain. The automatic power cycle of the devices is handled with a YKUSH USB (https://www.yepkit.com/products/ykush) hub, enabling the control of three USB ports power independently.

### Monitoring

To monitor the status of the platform at any given time, each packet contains a header with metadata about the command to execute and additional options. To ensure preventing Bit Flips during data transmission, PUF quality parameters being very sensitive to bit changes, every packet comprises a checksum calculated as the CRC16 of the packet. Additionally, since every operation the platform performs is atomic, any problems that may occur at any given moment can easily be located and reported. As an example, Table 2 portrays potential issues that could be detected while performing a WRITE operation on a device.

Fail-safe mechanism are introduced to detect more complex trouble and immediately stop the platform to secure data integrity. (i.e. a command could hang indefinitely waiting for lost bytes or part of the devices could remain inaccessible due to a physical problem in the chain).

### Database

A SQL database is used for reliable long term storage and effortless data filtering. Communication with the database is designed to be agnostic so any SQL database should work; for this project we have chosen PostgreSQL. The memory and sensors readouts are stored into two separate tables. Tables 3, 4 represent them. Along with sample information, a UTC timestamp is stored with each document to keep track of when the sample was extracted.

**Table 3:** CRPs schema.

id	Internal ID of the sample.
board_type	Type of device in the chain.
uid	Universal ID of the device.
pic	Position In Chain of the device.
address	Hex formatted region of SRAM.
data	List of values from memory.
created_at	UTC timestamp.

**Table 4:** Sensors schema.

id	Internal ID of the sample.
board_type	Type of device in the chain.
uid	Universal ID of the device.
temperature	Temperature of the device.
voltage	Voltage of the device.
created_at	UTC timestamp.

## Data Records

A static version of the full dataset available at the time of writing can be downloaded from Zenodo 10.5281/zenodo.7529513^10^. More data can be found online as explained in section 5 (Usage notes). The full dataset contains SRAM readouts of 84 Nucleo microcontrollers of STM32 type along with their voltage and temperature sensor data. This dataset is composed of two CSV files:

- The first one houses the readout of the SRAM memories. There are 9 samples per device and each memory is split into 160 regions of 512 bytes, summing up to a total of 120961 rows.
- The second files accommodates temperature and voltage sensor readouts. It contains 11 readouts, 9 of which were performed along with the SRAM memory readouts. There are a total of 925 rows.

The fields of both files are detailed in Tables 5, 6:

**Table 5:** CSV file housing SRAM memory readouts.

Field	Description
board_type	Device model that is connected to the chain. This dataset only contains Nucleo.
uid	STM32 96-bit ID formatted as 24 Hex-character string. This UID includes the batch number of the device, its wafer number, wafer lot and the X and Y position of the *μ*C on the wafer.
pic	The *Position In Chain*. Devices are assembled in a chain-like architecture to maximize their connections to the computer that performs the readout. The first device has a PIC of 1 and the last one has a PIC of 84.
address	The address of each memory region where the data was read. Each memory region contains exactly 512 bytes. For Nucleo devices, there are 160 regions in total. The SRAM memory of them starts at address 0 × 20000000.
data	512 bytes as unsigned integers of the memory region. The values are separated with commas and surrounded with double quotation marks.
created_at	Timestamp when data was gathered in ISO format (YYYY-MM-DD hh:mm:ss)

**Table 6:** CSV file housing temperature and voltage sensors.

Field	Description
board_type	*Identical to description in* Table 5.
uid	*Identical to description in* Table 5.
pic	*Identical to description in* Table 5.
temperature	Temperature of the device in Celsius.
voltage	Internal voltage of the device in Volts.
created_at	*Identical to description in* Table 5.

## Technical Validation

The data provided by this platform has not been pre-processed so as to keep raw data of the SRAM. Nevertheless, it is still important to ensure that the data provided does not present faulty bits and complies with the quality metrics depicted in the literature. Earlier, we have already detailed the different techniques of monitoring and error reporting used to guarantee the physical integrity of data Table 7.

**Table 7:** Table of request parameters and their accepted values.

Name	Possible values
Date	YYYY-MM-DD
Block	Positive integer
DateFrom-Day	1 to 30
DateFrom-Month	1 to 12
DateFrom-Year	4-digit year
DateTo-Day	1 to 31
DateTo-Month	1 to 12
DateTo-Year	4-digit year
BoardId	“all” or “0x” + 24-character UID + “−” + pic
CircuitId	“all” or “NUCLEO” or “DISCOVERY”

The minimum time required to assure that every device is correctly turned on or off is approximately 30 seconds. For every power cycle we have waited at least one minute; comfort interval to make sure that the SRAM contents are completely erased, knowing that an attacker could exploit data remanence and get PUF responses indirectly^11^.

Moreover, the quality of the samples has been studied with the canonical quality metrics published by Maiti *et al*.^2^. We will provide a summary of such metrics in order to assess the performance of SRAM on the *μ*Cs as a PUF. The purpose of these widely used metrics is to find vulnerabilities in the PUF behaviour such as bias in the distribution of 1 s and 0 s, systematic variation, etc. These metrics are updated every 24 hours. Nevertheless, the user can select an specific device and obtain its metrics in real time. For the metrics that require reference values of response bits (e.g. *reliability*), these values are derived by Majority Voting among samples. In the near future, we plan to include new metrics such as the ones presented in^12^.

**Fig. 2:**
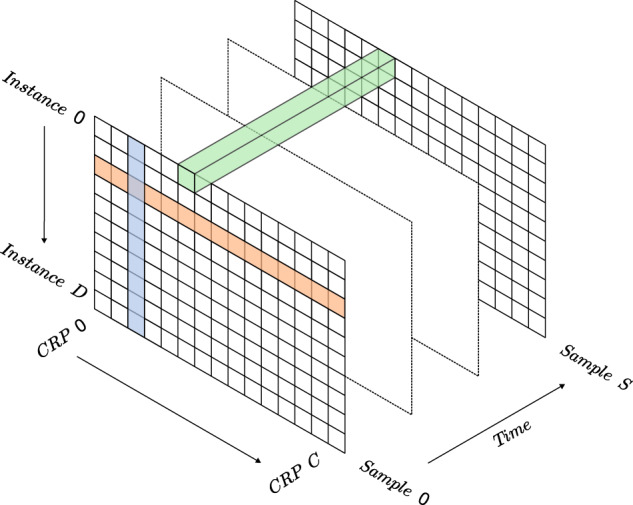
Diagram showing how data arrangement and calculations are performed. Orange: *uniformity*; blue: bit-aliasing; green: *reliability*; *uniqueness* is calculated for every combination of pair of rows. The metrics are monitored in real time in Grafana.

Before explaining the calculation of the metrics, a small visual aid on how calculations are performed is displayed in Fig. 2. Each row contains all the bits of each device and is arranged into a 3D matrix, where the third dimension gathers all the readouts of the devices.

### Uniformity

It measures the randomness of each device. It is calculated as the average of all responses of each device, meaning the average of the responses across rows. The following formula is used to calculate the uniformity of each device, where *C* represents the number of bits in the SRAM. This value should be 0.5 to ensure an even distribution of 0 s and 1 s.

1 $$Uniformity=\frac{1}{C}\mathop{\sum }\limits_{c=0}^{C}cr{p}_{c}$$

### Bit-aliasing

It assesses the randomness of each challenge across devices. It is calculated in the similar manner as *uniformity* but on columns instead of rows. The following formula is used for each challenge where *D* represents the number of devices that are studied. As with *uniformity*, this value should be 0.5.

2 $$Bit-aliasing=\frac{1}{D}\mathop{\sum }\limits_{c=0}^{D}cr{p}_{d}$$

### Uniqueness

This metric measures the difference in responses from pairs of devices, hoping there is enough randomness across devices. The *HD*_*norm*_ function refers to the normalized hamming distance and *C* to all the responses from a device in a given sample. The ideal value should be close to 0.5 to assure that each device produces a unique set of CRPs.

3 $$Uniqueness=\frac{1}{P}\mathop{\sum }\limits_{i=0}^{D}\mathop{\sum }\limits_{j=i+1}^{D}1-H{D}_{norm}\left({C}_{i},{C}_{j}\right)\quad {\rm{where}}\;P=\left(\begin{array}{l}D\\ 2\end{array}\right)$$

### Reliability

It determines the variability of responses in time and different conditions. The *reliability* of each device is calculated by grouping all responses into a vector and performing hamming distance with all the rest of the samples. The ideal value should be 1 to prove that responses do not change in time. It is important to mention that at least two samples are needed to calculate *reliability* as the hamming distance of a sample with itself is 0.

4 $$Reliability=1-\frac{1}{S}\sum _{s\in S}H{D}_{norm}\left({s}_{i},{s}_{ref}\right)$$

**Fig. 3:**
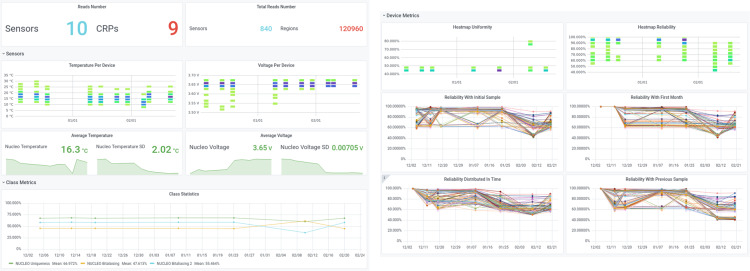
Snapshots of the Grafana dashboard used to monitor in real time the operation of the platform.

These quality metrics are measured when a new readout is performed and monitored through Grafana (https://grafana.com/) to asses potential problems during the readout. Figure 3 displays two snapshots of the Grafana dashboard checking the quality of the samples.

**Fig. 4:**
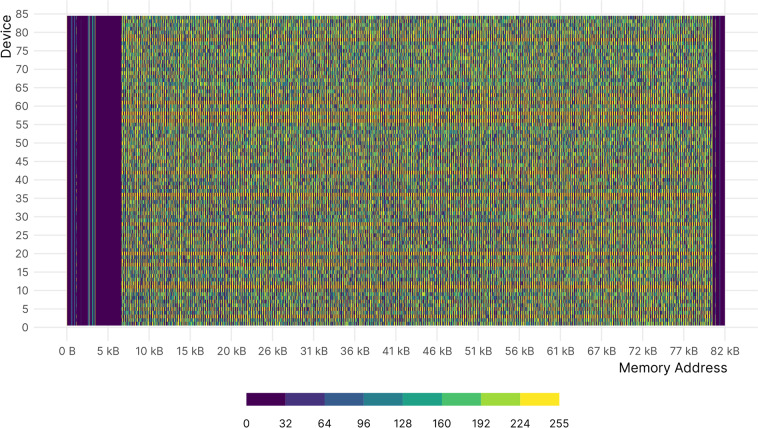
Heat map of a memory sample of 84 devices connected to the platform. Each tile refers to a byte of memory. Low regions of memory are represented in the left side and high regions are represented in the right.

Figure 4 shows a heat map of a memory sample of the 84 devices connected to the platform. Each row corresponds to a device and colors indicate the value of each memory cell. The areas at the beginning and at the end of each SRAM are full of 0 s as they are used for the stack to load the executable code and buffers. To secure the proper operation of the devices, these areas are read but not written. This heat map can be seen as one of the 2D matrix adopted to calculate metrics.

### Limitations

One of the main constraints of this platform is the acquisition time of the samples. The setup (described in the *Methods* section) follows a chain-like structure, the memory content is thus received and transmitted one after the other according to the position of the board in the chain. With 84 devices connected in one chain, it takes approximately 15 minutes to transmit the 80 kilobytes of the entire SRAM of the last board of the chain; therefore, the memory from all 84 devices would be retrieved in about 24 hours. In case of error during communication, a packet notifying the faulty bits is sent back to the PC and the information is retransmitted, with a consequent increase in transmission delay.

The other limitation is that the control of voltage and temperature is not feasable at the moment; the devices are subjected to environmental conditions.

## Usage Notes

Although the dataset described in this document is static, more data can be requested online through the following application hosted at https://puf4iot.univ-grenoble-alpes.fr/form.php. Any user can submit their need to the server specifying the data they want to retrieve, and a CSV or zip file (if larger records) will be handed over.

The database with raw data is updated once a week. The SRAM start-up values have been organized in memory regions of 512 bytes, proving a good trade-off between usability and data integrity. Each sample has been time-stamped in order to know the collection date.

**Fig. 5:**
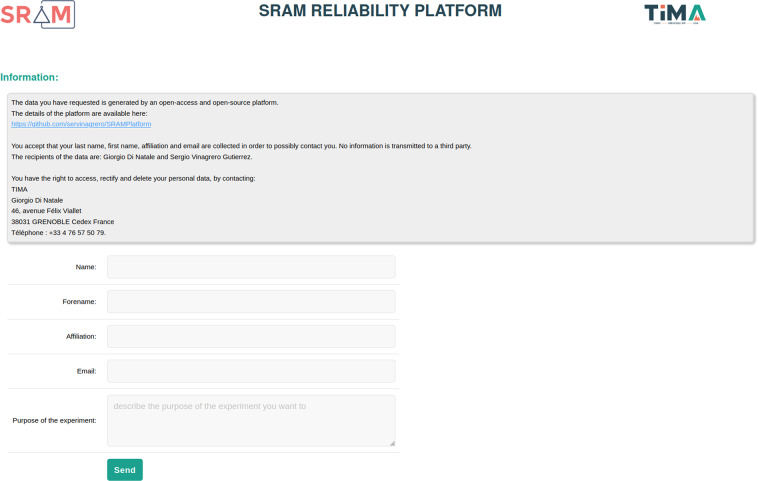
Form from PUF4IOT website displayed when requesting data.

Figure 5 pictures the entry point of the website. Users provide their information and are given access to the main site where they can ask for the needed data guided by filters. The website will then fetch the required data and generate the CSV or zip file.

Data can also be asked by making an HTTP request to the server with the filter parameters. Curl or Wget commands can be employed to get data directly from a terminal.

$ wget --post-data 'Param = value&Param2=value2...' https://puf4iot.univ-grenoble-alpes.fr/getdata.php

Table 7 shows all the possible request parameters and their accepted values.

When a vast amount of data is required, it will be split into various CSV files and contained in a zip, that can be saved with the following script; in this request, the Block parameter allows to skip certain number of records (here 2000).


#!/usr/bin/bashparams='Date=2020-01-22&BoardId=NUCLEO&CircuitId=0x3430716367336321B0660-1&Block=2'wget --post-data $params \


https://puf4iot.univ-grenoble-alpes.fr/getdata.php\ -O srampuf-tima-2020-01-22-NUCLEO-0x3430716367336321B0660-1-2.zip

Although SRAM and sensors data were earlier presented as being split into two different CSV files, data is merged when requested through the website. Therefore, every generated CSV file contains the following columns:


id;TimeStamp;BoardId;UDID;Position;MemAddress;Size;Temperature;Voltage;Response


## Data Availability

The source code of the platform and the STM32 devices are available under the GPL-2.0 license at https://github.com/servinagrero/SRAMPlatform. Online documentation on the platform and guidance on custom station set up can be found at https://servinagrero.github.io/SRAMPlatform. The full list of python dependencies is available in the *pyproject.toml* file in GitHub repository. PostgreSQL (https://www.postgresql.org/) is needed to store data, a message broker is necessary to communicate with the station, RabbitMQ (https://www.rabbitmq.com/)in our case, and Grafana is used to monitory metrics and sensors in a dashboard.
